# Supplementary material for: Hall sensors batch-fabricated on all-CVD h-BN/graphene/h-BN heterostructures
Source: Sci Rep. 2017 Nov 9;7:15231. doi: 10.1038/s41598-017-12277-8 (PMC5680335; doi:10.1038/s41598-017-12277-8)
Supplement: Supplementary file 1 — Supplementary Information [file 41598_2017_12277_MOESM1_ESM.pdf]

Supplementary materials

Hall sensors batch-fabricated on all-CVD h-BN/graphene/h-BN heterostructures

André Dankert[†*], Bogdan Karpiak[*], Saroj P. Dash[‡]

Department of Microtechnology and Nanoscience, Chalmers University of Technology, SE-41296, Göteborg, Sweden.

S1. Characterization of all-CVD 2D materials and heterostructures.

The 2D materials used in Hall sensor fabrication are grown by CVD method over large area. The CVD grown graphene samples were obtained from Graphenea and CVD h-BN from Graphene Supermarket. The Raman spectrum of graphene on SiO_2/Si substrate show the G and 2D peaks at 1597 cm^{-1} and 2652 cm^{-1} (Fig. S1a), with a small D peak[1]. At some areas bi-layer patches are observed. The grain size of the CVD graphene is mostly between 1-5 µm range.

Figure S1b shows Raman of multilayer h-BN with peak[2] at 1357 cm^{-1} for selective places. The peak typical for h-BN is not present at most of the areas, pointing to structural disorders. As already known from TEM characterization of such multilayer h-BN by Kim et al[3], the films are polycrystalline in nature and the c-axis of the crystallites points to random directions. The EELS studies by Kim et al. also indicated that the h-BN film is having sp^2 bonds and 1:1 stoichiometry of B and N atoms.

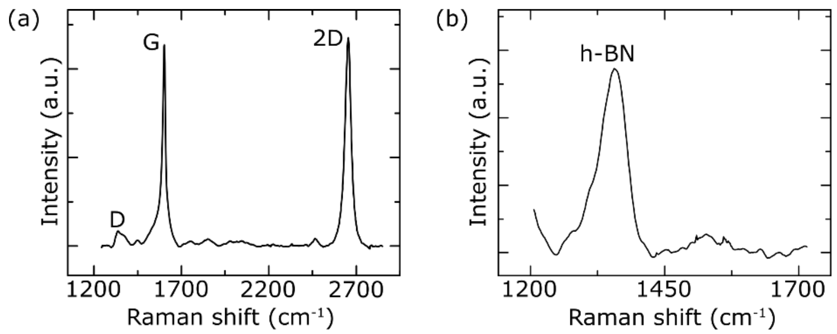

Supplementary Figure S1. *Raman spectra of (a) CVD graphene, (b) CVD h-BN.*

Corresponding authors: [†]andre.dankert@chalmers.se; [‡]saroj.dash@chalmers.se

* These authors contributed equally.

S2. AFM of h-BN after annealing

To investigate the influence of annealing on the quality of CVD h-BN, the sample with h-BN after transfer on Si/SiO_2 substrate was put in a furnace at 400 ℃ in Ar/H_2 atmosphere for 8 hours. After the annealing, the topography of the surface was investigated by AFM (Fig. S2). The surface roughness of CVD h-BN was found to be ~2 nm, similar to the one before annealing (see Fig. 2b in the main text).

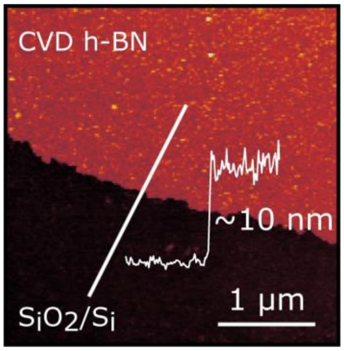

Supplementary Figure S2. *AFM image and thickness profile of CVD h-BN on SiO_2/Si wafer after annealing.*

S3. Magnetic resolution of all-CVD h-BN/graphene/h-BN Hall sensors

From noise spectral measurements on fully-encapsulated graphene Hall sensor with Cr/Au contacts (Fig. S3) the minimum magnetic resolution ($B_{min}=S_V^{0.5}/(S_I I)$, where S_V is the noise power spectral density) was found to be 0.4 mT/$Hz^{0.5}$ at frequency of 1 kHz, which is significantly lower in comparison to previous reports. Such difference could be attributed to the quality of h-BN/graphene/h-BN heterostructures after large-area wet transfer process from the CVD growth substrate.

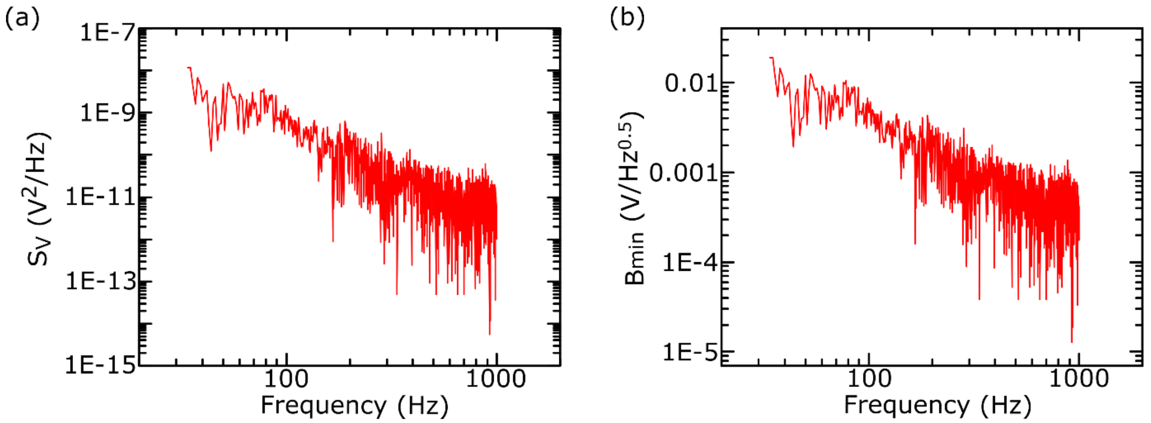

Supplementary Figure S3. *Noise characterization of all-CVD h-BN/graphene/h-BN Hall sensors. (a) Noise power spectral density S_V and (b) minimum magnetic resolution B_{min}.*

S4. Graphene Hall sensors prepared on SiO_2 substrate with and without exfoliated h-BN encapsulation

To compare the performance of Hall sensors fabricated on large area all-CVD heterostructures prepared by wet-transfer technique, we fabricated another control samples with CVD graphene on Si/SiO_2 substrate with and without top encapsulation by exfoliated h-BN (Fig. S4). From the Hall measurements (Figs. S3b and S3d) the Hall mobility was found to be around 150 cm^2/Vs^{-1}, hole doping concentration ~10^{12} cm^{-2}. The current-related sensitivity S_I in device with h-BN encapsulation (363 V/AT) was found to be 3 to 4 times higher than in all-CVD heterostructure-based Hall sensors, while unencapsulated devices showed S_I in the range 100-200 V/AT after fabrication, degrading down to 50 V/AT after two months.

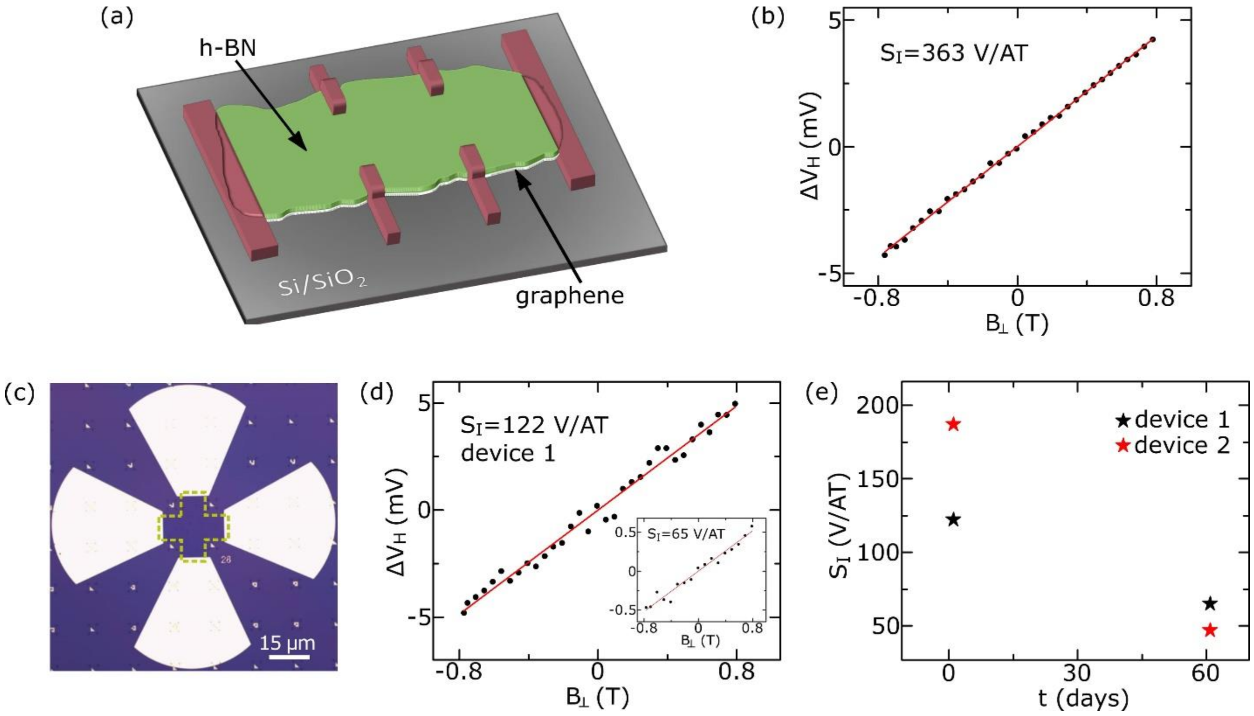

Supplementary Figure S4. *CVD graphene Hall sensors fabricated on Si/SiO_2 substrate. (a) Schematic representation of the device with encapsulation by exfoliated h-BN flakes. (b) Hall voltage response of the device as a function of perpendicular magnetic field at room temperature (background is subtracted). (c) Optical microscope picture of the device without encapsulation. Graphene edges are marked by yellow dashed line. (d) Hall voltage response of the new unencapsulated device (main panel) and 2 months after fabrication (inset). Linear background is subtracted. (e) Time-dependent current-related sensitivity plot for the two unencapsulated CVD graphene samples.*

References

1. Ferrari, A. ., Meyer, J. C., Scardaci, C., Casiraghi, C. & Lazzeri, M. Raman Spectrum of Graphene and Graphene Layers. *Phys. Rev. Lett.* **97,** 187401 (2006).

2. Gorbachev, R. V. *et al.* Hunting for monolayer boron nitride: Optical and raman signatures. *Small* **7,** 465–468 (2011).

3. Kim, K. K. *et al.* Synthesis and Characterization of Hexagonal Boron Nitride Film as a Dielectric Layer for Graphene Devices. *ACS Nano* **6,** 8583–8590 (2012).
